# The Stroop Color and Word Test

**DOI:** 10.3389/fpsyg.2017.00557

**Published:** 2017-04-12

**Authors:** Federica Scarpina, Sofia Tagini

**Affiliations:** ^1^“Rita Levi Montalcini” Department of Neuroscience, University of TurinTurin, Italy; ^2^IRCCS Istituto Auxologico Italiano, Ospedale San GiuseppePiancavallo, Italy; ^3^CiMeC Center for the Mind/Brain Sciences, University of TrentoRovereto, Italy

**Keywords:** stroop color and word test, neuropsychological assessment, inhibition, executive functions, systematic review

## Abstract

The Stroop Color and Word Test (SCWT) is a neuropsychological test extensively used to assess the ability to inhibit cognitive interference that occurs when the processing of a specific stimulus feature impedes the simultaneous processing of a second stimulus attribute, well-known as the Stroop Effect. The aim of the present work is to verify the theoretical adequacy of the various scoring methods used to measure the Stroop effect. We present a systematic review of studies that have provided normative data for the SCWT. We referred to both electronic databases (i.e., PubMed, Scopus, Google Scholar) and citations. Our findings show that while several scoring methods have been reported in literature, none of the reviewed methods enables us to fully assess the Stroop effect. Furthermore, we discuss several normative scoring methods from the Italian panorama as reported in literature. We claim for an alternative scoring method which takes into consideration both speed and accuracy of the response. Finally, we underline the importance of assessing the performance in all Stroop Test conditions (word reading, color naming, named color-word).

## Introduction

The Stroop Color and Word Test (SCWT) is a neuropsychological test extensively used for both experimental and clinical purposes. It assesses the ability to inhibit cognitive interference, which occurs when the processing of a stimulus feature affects the simultaneous processing of another attribute of the same stimulus (Stroop, 1935). In the most common version of the SCWT, which was originally proposed by Stroop in the 1935, subjects are required to read three different tables as fast as possible. Two of them represent the “congruous condition” in which participants are required to read names of colors (henceforth referred to as color-words) printed in black ink (W) and name different color patches (C). Conversely, in the third table, named color-word (CW) condition, color-words are printed in an inconsistent color ink (for instance the word “red” is printed in green ink). Thus, in this incongruent condition, participants are required to name the color of the ink instead of reading the word. In other words, the participants are required to perform a less automated task (i.e., naming ink color) while inhibiting the interference arising from a more automated task (i.e., reading the word; MacLeod and Dunbar, 1988; Ivnik et al., 1996). This difficulty in inhibiting the more automated process is called the Stroop effect (Stroop, 1935). While the SCWT is widely used to measure the ability to inhibit cognitive interference; previous literature also reports its application to measure other cognitive functions such as attention, processing speed, cognitive flexibility (Jensen and Rohwer, 1966), and working memory (Kane and Engle, 2003). Thus, it may be possible to use the SCWT to measure multiple cognitive functions.

In the present article, we present a systematic review of the SCWT literature in order to assess the theoretical adequacy of the different scoring methods proposed to measure the Stroop effect (Stroop, 1935). We focus on Italian literature, which reports the use of several versions of the SCWT that vary in in terms of stimuli, administration protocol, and scoring methods. Finally, we attempt to indicate a score method that allows measuring the ability to inhibit cognitive interference in reference to the subjects' performance in SCWT.

## Methods

We looked for normative studies of the SCWT. All studies included a healthy adult population. Since our aim was to understand the various available scoring methods, no studies were excluded on the basis of age, gender, and/or education of participants, or the specific version of SCWT used (e.g., short or long, computerized or paper). Studies were identified using electronic databases and citations from a selection of relevant articles. The electronic databases searched included PubMed (All years), Scopus (All years) and Google Scholar (All years). The last search was run on the 22nd February, 2017, using the following search terms: “Stroop; test; normative.” All studies written in English and Italian were included.

Two independent reviewers screened the papers according to their titles and abstracts; no disagreements about suitability of the studies was recorded. Thereafter, a summary chart was prepared to highlight mandatory information that had to be extracted from each report (see Table 1).

**Table 1:** **Summary of data extracted from reviewed articles; those related to the Italian normative data are in bold**.

**References**	**Index**
Ingraham et al., 1988; Ivnik et al., 1996; Rosselli et al., 2002; Moering et al., 2004; Lucas et al., 2005; Steinberg et al., 2005; Seo et al., 2008; Peña-Casanova et al., 2009; Al-Ghatani et al., 2011; Norman et al., 2011; Andrews et al., 2012; Llinàs-Reglà et al., 2013; Morrow, 2013; Lubrini et al., 2014; Rivera et al., 2015; Waldrop-Valverde et al., 2015	IG = CW − [(W × C)/(W + C)] where IG: interference score; CW: number of items properly named in 45 s in the CW condition; W: number of items properly named in 45 s in the W condition; C: number of items properly named in 45 s in the C condition.
Troyer et al., 2006; Bayard et al., 2011; Campanholo et al., 2014; Bezdicek et al., 2015; Hankee et al., 2016; Tremblay et al., 2016	Completion time for each condition. Number of errors (corrected, not corrected, total errors) in each condition. Low Interference score: W/C where W: time to read commons words printed in different colored ink; C: time to name colored dots. High Interference score: CW/C where CW: time to read colors names printed in incongruent colored ink; C: time to name colored dots.
Strickland et al., 1997; Kang et al., 2013	Time completion in W, C and CW condition. Errors in W, C, and CW condition.
Amato et al., 2006	Time to name 50 items in the CW condition.
Barbarotto et al., 1998	Correct answers in 30 s in C and in CW condition. Shortest interval (in seconds) of the sequence correctly read in C and CW condition.
Brugnolo et al., 2015	Correct answers in 30 s in W, C, and CW condition. T to read the table in W, C, and CW condition.
Caffarra et al., 2002	TI = CWT − [(WT + CT)/2] where TI: time interference score; WT: time to complete W condition; CT: time to complete C condition; CWT: time to complete CW condition. EI = CWE − [(WE + CE)/2] Where EI: error interference score; EI: errors interference score; WE: errors in W condition; CE: errors in C condition; CWE: errors in CW condition.
Valgimigli et al., 2010	I = [(DC − DI)/(DC + DI)] × 100 where DC: correct answers in 20 s in C condition; DI: correct answers in 20 s in CW condition.
Van der Elst et al., 2006	Time to complete W, C, and CW conditions. Number of errors not self-corrected in W, C, and CW conditions. Interference score: TI = CWT − [(WT + CT)/2] where TI: time interference score; WT: time to complete W condition; CT: time to complete C condition; CWT: time to complete CW condition.
Zalonis et al., 2009	Time to read 112 words of colors printed in incongruous colored ink. Number of errors and number of self-corrections in the CW condition. Interference score for the CW condition: Number of items properly named in 120 s—number of errors.
Zimmermann et al., 2015	Errors in W, C, and CW condition. Corrected answer in 45 s in W, C, and CW, condition. Interference score: Time to read CW + [errors CW × 2(time to read CW/number of items in CW)].

One Author extracted data from papers while the second author provided further supervision. No disagreements about extracted data emerged. We did not seek additional information from the original reports, except for Caffarra et al. (2002), whose full text was not available: relevant information have been extracted from Barletta-Rodolfi et al. (2011).

We extracted the following information from each article:

- Year of publication.
- Indexes whose normative data were provided.

Eventually, as regards the variables of interest, we focused on those scores used in the reviewed studies to assess the performance at the SCWT.

## Results

**Figure 1:**
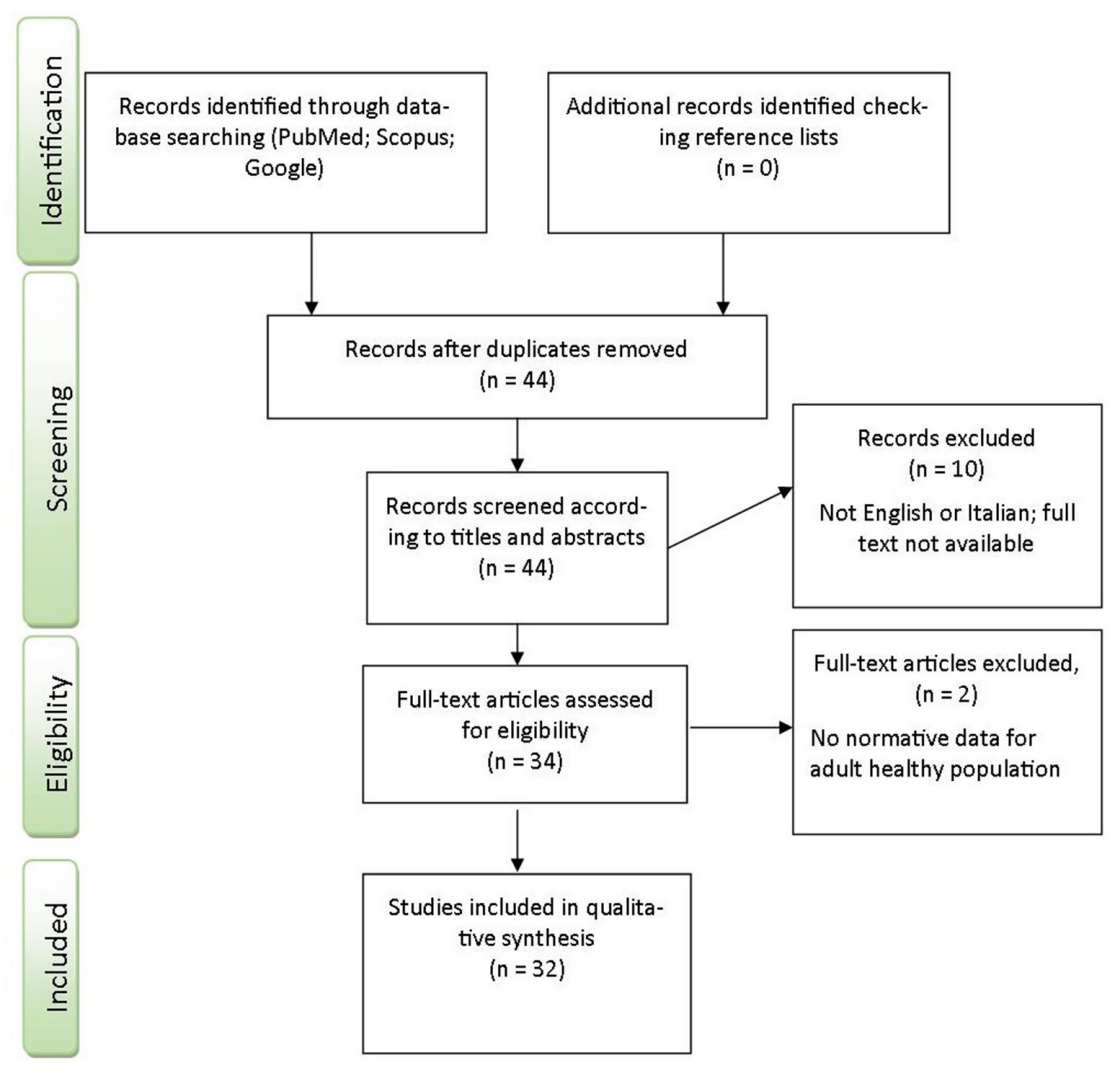
**Flow diagram of studies selection process**.

We identified 44 articles from our electronic search and screening process. Eleven of them were judged inadequate for our purpose and excluded. Four papers were excluded as they were written in languages other than English or Italian (Bast-Pettersen, 2006; Duncan, 2006; Lopez et al., 2013; Rognoni et al., 2013); two were excluded as they included children (Oliveira et al., 2016) and a clinical population (Venneri et al., 1992). Lastly, we excluded six Stroop Test manuals, since not entirely procurable (Trenerry et al., 1989; Artiola and Fortuny, 1999; Delis et al., 2001; Golden and Freshwater, 2002; Mitrushina et al., 2005; Strauss et al., 2006a). At the end of the selection process we had 32 articles suitable for review (Figure 1).

From the systematic review, we extracted five studies with Italian normative data. Details are reported in Table 1. Of the remaining 27 studies that provide normative data for non-Italian populations, 16 studies (Ivnik et al., 1996; Ingraham et al., 1988; Rosselli et al., 2002; Moering et al., 2004; Lucas et al., 2005; Steinberg et al., 2005; Seo et al., 2008; Peña-Casanova et al., 2009; Al-Ghatani et al., 2011; Norman et al., 2011; Andrews et al., 2012; Llinàs-Reglà et al., 2013; Morrow, 2013; Lubrini et al., 2014; Rivera et al., 2015; Waldrop-Valverde et al., 2015) adopted the scoring method proposed by Golden (1978). In this method, the number of items correctly named in 45 s in each conditions is calculated (i.e., W, C, CW). Then the predicted CW score (Pcw) is calculated using the following formula:

(1) $$\begin{array}{r} \text{Pcw }=\;45/\left\{\left(\left(45\;\times \text{ W}\right)\;+\;\left(45\;\times \text{ C}\right)\right)/\left(\text{W }\times \text{ C}\right)\right\} \end{array}$$

equivalent to:

(2) $$\begin{array}{r} \text{Pcw }=\;\left(\text{W }\times \text{ C}\right)/\left(\text{W }+\text{ C}\right) \end{array}$$

Then, the Pcw value is subtracted from the actual number of items correctly named in the incongruous condition (CW) (i.e., IG = CW − Pcw): this procedure allows to obtain an interference score (IG) based on the performance in both W and C conditions. Thus, a negative IG value represents a pathological ability to inhibit interference, where a lower score means greater difficulty in inhibiting interference.

Six articles (Troyer et al., 2006; Bayard et al., 2011; Campanholo et al., 2014; Bezdicek et al., 2015; Hankee et al., 2016; Tremblay et al., 2016) adopted the Victoria Stroop Test. In this version, three conditions are assessed: the C and the CW correspond to the equivalent conditions of the original version of the test (Stroop, 1935), while the W condition includes common words which do not refer to colors. This condition represents an intermediate inhibition condition, as the interference effect between the written word and the color name is not present. In this SCWT form (Strauss et al., 2006b), for each condition, the completion time and the number of errors (corrected, non-corrected, and total errors) are recorded and two interference scores are computed:

(3) $$\begin{array}{r} \text{I}1\;=\text{ Word}/\text{Dot for time} \end{array}$$

(4) $$\begin{array}{r} \text{I}2\;=\text{ Interference}/\text{Dot for time} \end{array}$$

Five studies (Strickland et al., 1997; Van der Elst et al., 2006; Zalonis et al., 2009; Kang et al., 2013; Zimmermann et al., 2015) adopted different SCWT versions. Three of them (Strickland et al., 1997; Van der Elst et al., 2006; Kang et al., 2013) computed, independently, the completion time and the number of errors for each condition. Additionally, Van der Elst et al. (2006), computed an interference score based on the speed performance only:

(5) $$\begin{array}{r} \text{TI }=\text{ CWT }-\;\left[\left(\text{WT }+\text{ CT}\right)/2\right] \end{array}$$

where WT, CT, and CWT represent the time to complete the W, C, and CW table, respectively. Zalonis et al. (2009) recorded: (i) the time; (ii) the number of errors and (iii) the number of self-corrections in the CW. Moreover, they computed an interference score subtracting the number of errors in the CW conditions from the number of items properly named in 120 s in the same table. Lastly, Zimmermann et al. (2015) computed the number of errors and the number of correct answers given in 45 s in each conditions. Additionally, they calculated an interference score derived by the original scoring method provided by Stroop (1935).

Of the five studies (Barbarotto et al., 1998; Caffarra et al., 2002; Amato et al., 2006; Valgimigli et al., 2010; Brugnolo et al., 2015) that provide normative data for the Italian population, two are originally written in Italian (Caffarra et al., 2002; Valgimigli et al., 2010), while the others are written in English (Barbarotto et al., 1998; Amato et al., 2006; Brugnolo et al., 2015). An English translation of the title and abstract of Caffarra et al. (2002) is available. Three of the studies consider the performance only on the SCWT (Caffarra et al., 2002; Valgimigli et al., 2010; Brugnolo et al., 2015) while the others also include other neuropsychological tests in the experimental assessment (Barbarotto et al., 1998; Amato et al., 2006). The studies are heterogeneous in that they differ in terms of administered conditions, scoring procedures, number of items, and colors used. Three studies adopted a 100-items version of the SCWT (Amato et al., 2006; Valgimigli et al., 2010; Brugnolo et al., 2015) which is similar to the original version proposed by Stroop (1935). In this version, in every condition (i.e., W, C, CW), items are arranged in a matrix of 10 × 10 columns and rows; the colors are red, green, blue, brown, and purple. However, while two of these studies administered the W, C, and CW conditions once (Amato et al., 2006; Valgimigli et al., 2010), Barbarotto et al. (1998) administered the CW table twice, requiring participants to read the word during the first administration and then to name the ink color during the consecutive administration. Additionally, they also administered a computerized version of the SCWT in which 40 stimuli are presented in each condition; red, blue, green, and yellow are used. Valgimigli et al. (2010) and Caffarra et al. (2002) administered shorter paper versions of the SCWT including only three colors (i.e., red, blue, green). More specifically, the former administered only the C and CW conditions including 60 items each, arranged in six columns of 10 items. The latter employed a version of 30 items for each condition (i.e., W, C, CW), arranged in three columns of 10 items each.

Only two of the five studies assessed and provided normative data for all the conditions of the SCWT (i.e., W, C, CW; Caffarra et al., 2002; Brugnolo et al., 2015), while others provide only partial results. Valgimigli et al. (2010) provided normative data only for the C and CW condition, while Amato et al. (2006) and Barbarotto et al. (1998) administered all the SCWT conditions (i.e., W, C, CW) but provide normative data only for the CW condition, and the C and CW condition respectively.

These studies use different methods to compute subjects' performance. Some studies record the time needed, independently in each condition, to read all (Amato et al., 2006) or a fixed number (Valgimigli et al., 2010) of presented stimuli. Others consider the number of correct answers produced in a fixed time (30 s; Amato et al., 2006; Brugnolo et al., 2015). Caffarra et al. (2002) and Valgimigli et al. (2010) provide a more complex interference index that relates the subject's performance in the incongruous condition with the performance in the others. In Caffarra et al. (2002), two interference indexes based on reading speed and accuracy, respectively, are computed using the following formula:

(6) $$\begin{array}{r} \text{I }=\text{ CW }-\;\left(\left(\text{W }+\text{ C}\right)/2\right) \end{array}$$

Furthermore, in Valgimigli et al. (2010) an interference score is computed using the formula:

(7) $$\begin{array}{r} \text{I }=\;\left(\left(\text{DC }-\text{ DI}\right)/\left(\text{DC }+\text{ DI}\right)\right)\;\times \;100 \end{array}$$

where DC represents the correct answers produced in 20 s in naming colors and DI corresponds to the correct answers achieved in 20 s in the interference condition. However, they do not take into account the performance on the word reading condition.

## Discussion

According to the present review, multiple SCWT scoring methods are available in literature, with Golden's (1978) version being the most widely used. In the Italian literature, the heterogeneity in SCWT scoring methods increases dramatically. The parameters of speed and accuracy of the performance, essential for proper detection of the Stroop Effect, are scored differently between studies, thus highlighting methodological inconsistencies. Some of the reviewed studies score solely the speed of the performance (Amato et al., 2006; Valgimigli et al., 2010). Others measure both the accuracy and speed of performance (Barbarotto et al., 1998; Brugnolo et al., 2015); however, they provide no comparisons between subjects' performance on the different SCWT conditions. On the other hand, Caffarra et al. (2002) compared performance in the W, C, and CW conditions; however, they computed speed and accuracy independently. Only Valgimigli et al. (2010) present a scoring method in which an index merging speed and accuracy is computed for the performance in all the conditions; however, the Authors assessed solely the performance in the C and the CW conditions, neglecting the subject's performance in the W condition.

In our opinion, the reported scoring methods impede an exhaustive description of the performance on the SCWT, as suggested by clinical practice. For instance, if only the reading time is scored, while accuracy is not computed (Amato et al., 2006) or is computed independently (Caffarra et al., 2002), the consequences of possible inhibition difficulties on the processing speed cannot be assessed. Indeed, patients would report a non-pathological reading speed in the incongruous condition, despite extremely poor performance, even if they do not apply the rule “naming ink color,” simply reading the word (e.g., in CW condition, when the stimulus is the word/red/printed in green ink, patient says “Red” instead of “Green”). Such behaviors provide an indication of the failure to maintain consistent activation of the intended response in the incongruent Stroop condition, even if the participants properly understand the task. Such scenarios are often reported in different clinical populations. For example, in the incongruous condition, patients with frontal lesions (Vendrell et al., 1995; Stuss et al., 2001; Swick and Jovanovic, 2002) as well as patients affected by Parkinson's Disease (Fera et al., 2007; Djamshidian et al., 2011) reported significant impairments in terms of accuracy, but not in terms of processing speed. Counting the number of correct answers in a fixed time (Amato et al., 2006; Valgimigli et al., 2010; Brugnolo et al., 2015) may be a plausible solution.

Moreover, it must be noted that error rate (and not the speed) is an index of inhibitory control (McDowd et al., 1995) or an index of ability to maintain the tasks goal temporarily in a highly retrievable state (Kane and Engle, 2003). Nevertheless, computing exclusively the error rate (i.e., the accuracy in the performance), without measuring the speed of performance, would be insufficient for an extensive evaluation of the performance in the SCWT. In fact, the behavior in the incongruous condition (i.e., CW) may be affected by difficulties that are not directly related to an impaired ability to suppress the interference process, which may lead to misinterpretation of the patient's performance. People affected by color-blindness or dyslexia would represent the extreme case. Nonetheless, and more ordinarily, slowness, due to clinical circumstances like dysarthria, mood disorders such as depression, or collateral medication effect, may irremediably affect the performance in the SCWT. In Parkinson's Disease, ideomotor slowness (Gardner et al., 1959; Jankovic et al., 1990) impacts the processing speed in all SCWT conditions, determining a global difficulty in the response execution rather than a specific impairment in the CW condition (Stacy and Jankovic, 1992; Hsieh et al., 2008). Consequently, it seems necessary to relate the performance in the incongruous condition to word reading and color naming abilities, when inhibition capability has to be assessed, as proposed by Caffarra et al. (2002). In this method the W score and C score were subtracted from CW score. However, as previously mentioned, the scoring method suggested by Caffarra et al. (2002) computes errors and speed separately. Thus, so far, none of the proposed Italian normative scoring methods seem adequate to assess patients' performance in the SCWT properly and informatively.

Examples of more suitable interference scores can be found in non-Italian literature. Stroop (1935) proposed that the ability to inhibit cognitive interference can be measured in the SCWT using the formula:

(8) $$\begin{array}{l} \text{total time }+\;((2\;\times \text{ mean time per word})\\ \times \text{ number of uncorrected errors}) \end{array}$$

where, total time is the overall time for reading; mean time per word is the overall time for reading divided by the number of items; and the number of uncorrected errors is the number of errors not spontaneously corrected. Gardner et al. (1959) also propose a similar formula:

(9) $$\begin{array}{r} \text{total time }+\;\left(\left(\text{total time}/100\right)\;\times \text{ number of errors}\right) \end{array}$$

where 100 refers to the number of stimuli used in this version of the SCWT. When speed and errors are computed together, the correct recognition of patients who show difficulties in inhibiting interference despite a non-pathological reading time, increases. However, both the mentioned scores (Stroop, 1935; Mitrushina et al., 2005) may be susceptible to criticism (Jensen and Rohwer, 1966). In fact, even though accuracy and speed are merged into a global score in these studies (Stroop, 1935; Mitrushina et al., 2005), they are not computed independently. In Gardner et al. (1959) the number of errors are computed in relation to the mean time *per* item and then added to the total time, which may be redundant and lead to a miscomputation.

The most adopted scoring method in the international panorama is Golden (1978). Lansbergen et al. (2007) point out that the index IG might not be adequately corrected for inter-individual differences in the reading ability, despite its effective adjustment for color naming. The Authors highlight that the reading process is more automated in expert readers, and, consequently, they may be more susceptible to interference (Lansbergen et al., 2007), thus, requiring that the score is weighted according to individual reading ability. However, experimental data suggests that the increased reading practice does not affect the susceptibility to interference in SCWT (Jensen and Rohwer, 1966). Chafetz and Matthews (2004)'s article might be useful for a deeper understanding of the relationship between reading words and naming colors, but the debate about the role of reading ability on the inhibition process is still open. The issue about the role of reading ability on the SCWT performance cannot be adequately satisfied even if the Victoria Stroop Test scoring method (Strauss et al., 2006b) is adopted, since the absence of the standard W condition.

In the light of the previous considerations, we recommend that a scoring method for the SCWT should fulfill two main requirements. First, *both* accuracy and speed must be computed for *all* SCWT conditions. And secondly, a global index must be calculated to relate the performance in the incongruous condition to reading words and color naming abilities. The first requirement can be achieved by counting the number of correct answers in each condition in within a fixed time (Amato et al., 2006; Valgimigli et al., 2010; Brugnolo et al., 2015). The second requirement can be achieved by subtracting the W score and C score from CW score, as suggested by Caffarra et al. (2002). None of the studies reviewed satisfies both these requirements.

According to the review, the studies with Italian normative data present different theoretical interpretations of the SCWT scores. Amato et al. (2006) and Caffarra et al. (2002) describe the SCWT score as a measure of the fronto-executive functioning, while others use it as an index of the attentional functioning (Barbarotto et al., 1998; Valgimigli et al., 2010) or of general cognitive efficiency (Brugnolo et al., 2015). Slowing to a response conflict would be due to a failure of selective attention or a lack in the cognitive efficiency instead of a failure of response inhibition (Chafetz and Matthews, 2004); however, the performance in the SCWT is not exclusively related to concentration, attention or cognitive effectiveness, but it relies to a more specific executive-frontal domain. Indeed, subjects have to process selectively a specific visual feature blocking out continuously the automatic processing of reading (Zajano and Gorman, 1986; Shum et al., 1990), in order to solve correctly the task. The specific involvement of executive processes is supported by clinical data. Patients with anterior frontal lesions, and not with posterior cerebral damages, report significant difficulties in maintaining a consistent activation of the intended response (Valgimigli et al., 2010). Furthermore, Parkinson's Disease patients, characterized by executive dysfunction due to the disruption of dopaminergic pathway (Fera et al., 2007), reported difficulties in SCWT despite unimpaired attentional abilities (Fera et al., 2007; Djamshidian et al., 2011).

## Conclusion

According to the present review, the heterogeneity in the SCWT scoring methods in international literature, and most dramatically in Italian literature, seems to require an innovative, alternative and unanimous scoring system to achieve a more proper interpretation of the performance in the SCWT. We propose to adopt a scoring method in which (i) the number of correct answers in a fixed time in each SCWT condition (W, C, CW) and (ii) a global index relative to the CW performance minus reading and/or colors naming abilities, are computed. Further studies are required to collect normative data for this scoring method and to study its applicability in clinical settings.

## Author contributions

Conception of the work: FS. Acquisition of data: ST. Analysis and interpretation of data for the work: FS and ST. Writing: ST, and revising the work: FS. Final approval of the version to be published and agreement to be accountable for all aspects of the work: FS and ST.

### Conflict of interest statement

The authors declare that the research was conducted in the absence of any commercial or financial relationships that could be construed as a potential conflict of interest.

## References

[B1] Al-Ghatani A. M. Obonsawin M. C. Binshaig B. A. Al-Moutaery K. R. (2011). Saudi normative data for the Wisconsin Card Sorting test, Stroop test, test of non-verbal intelligence-3, picture completion and vocabulary (subtest of the wechsler adult intelligence scale-revised). Neurosciences 16, 29–41. 21206442

[B2] Amato M. P. Portaccio E. Goretti B. Zipoli V. Ricchiuti L. De Caro M. F.. (2006). The Rao's brief repeatable battery and stroop test: normative values with age, education and gender corrections in an Italian population. Mult. Scler. 12, 787–793. 10.1177/1352458506070933 17263008

[B3] Andrews K. Shuttleworth-Edwards A. Radloff S. (2012). Normative indications for Xhosa speaking unskilled workers on the Trail Making and Stroop Tests. J. Psychol. Afr. 22, 333–341. 10.1080/14330237.2012.10820538

[B4] Artiola L. Fortuny L. A. I. (1999). Manual de Normas Y Procedimientos Para la Bateria Neuropsicolog. Tucson, AZ: Taylor & Francis.

[B5] Barbarotto R. Laiacona M. Frosio R. Vecchio M. Farinato A. Capitani E. (1998). A normative study on visual reaction times and two Stroop colour-word tests. Neurol. Sci. 19, 161–170. 10.1007/BF00831566 10933471

[B6] Barletta-Rodolfi C. Gasparini F. Ghidoni E. (2011). Kit del Neuropsicologo Italiano. Bologna: Società Italiana di Neuropsicologia.

[B7] Bast-Pettersen R. (2006). The Hugdahl Stroop Test: A normative stud y involving male industrial workers. J. Norwegian Psychol. Assoc. 43, 1023–1028.

[B8] Bayard S. Erkes J. Moroni C. (2011). Collège des psychologues cliniciens spécialisés en neuropsychologie du languedoc roussillon (CPCN Languedoc Roussillon). Victoria Stroop Test: normative data in a sample group of older people and the study of their clinical applications in the assessment of inhibition in Alzheimer's disease. Arch. Clin. Neuropsychol. 26, 653–661. 10.1093/arclin/acr053 21873625

[B9] Bezdicek O. Lukavsky J. Stepankova H. Nikolai T. Axelrod B. N. Michalec J.. (2015). The Prague Stroop Test: normative standards in older Czech adults and discriminative validity for mild cognitive impairment in Parkinson's disease. J. Clin. Exp. Neuropsychol. 37, 794–807. 10.1080/13803395.2015.1057106 26313510

[B10] Brugnolo A. De Carli F. Accardo J. Amore M. Bosia L. E. Bruzzaniti C.. (2015). An updated Italian normative dataset for the Stroop color word test (SCWT). Neurol. Sci. 37, 365–372. 10.1007/s10072-015-2428-2 26621362

[B11] Caffarra P. Vezzaini G. Dieci F. Zonato F. Venneri A. (2002). Una versione abbreviata del test di Stroop: dati normativi nella popolazione italiana. Nuova Rivis. Neurol. 12, 111–115.

[B12] Campanholo K. R. Romão M. A. Machado M. A. D. R. Serrao V. T. Coutinho D. G. C. Benute G. R. G. (2014). Performance of an adult Brazilian sample on the Trail Making Test and Stroop Test. Dement. Neuropsychol. 8, 26–31. 10.1590/S1980-57642014DN81000005 PMC5619445 29213876

[B13] Chafetz M. D. Matthews L. H. (2004). A new interference score for the Stroop test. Arch. Clin. Neuropsychol. 19, 555–567. 10.1016/j.acn.2003.08.004 15163456

[B14] Delis D. C. Kaplan E. Kramer J. H. (2001). Delis-Kaplan Executive Function System (D-KEFS). San Antonio, TX: Psychological Corporation.

[B15] Djamshidian A. O'Sullivan S. S. Lees A. Averbeck B. B. (2011). Stroop test performance in impulsive and non impulsive patients with Parkinson's disease. Parkinsonism Relat. Disord. 17, 212–214. 10.1016/j.parkreldis.2010.12.014 21247790 PMC3042030

[B16] Duncan M. T. (2006). Assessment of normative data of Stroop test performance in a group of elementary school students Niterói. J. Bras. Psiquiatr. 55, 42–48. 10.1590/S0047-20852006000100006

[B17] Fera F. Nicoletti G. Cerasa A. Romeo N. Gallo O. Gioia M. C.. (2007). Dopaminergic modulation of cognitive interference after pharmacological washout in Parkinson's disease. Brain Res. Bull. 74, 75–83. 10.1016/j.brainresbull.2007.05.009 17683792

[B18] Gardner R. W. Holzman P. S. Klein G. S. Linton H. P. Spence D. P. (1959). Cognitive control: a study of individual consistencies in cognitive behaviour. Psychol. Issues 1, 1–186.

[B19] Golden C. J. (1978). Stroop Color and Word Test: A Manual for Clinical and Experimental Uses. Chicago, IL: Stoelting Co.

[B20] Golden C. J. Freshwater S. M. (2002). The Stroop Color and Word Test: A Manual for Clinical and Experimental Uses. Chicago, IL: Stoelting.

[B21] Hankee L. D. Preis S. R. Piers R. J. Beiser A. S. Devine S. A. Liu Y.. (2016). Population normative data for the CERAD word list and Victoria Stroop Test in younger-and middle-aged adults: cross-sectional analyses from the framingham heart study. Exp. Aging Res. 42, 315–328. 10.1080/0361073X.2016.1191838 27410241 PMC4946576

[B22] Hsieh Y. H. Chen K. J. Wang C. C. Lai C. L. (2008). Cognitive and motor components of response speed in the Stroop test in Parkinson's disease patients. Kaohsiung J. Med. Sci. 24, 197–203. 10.1016/S1607-551X(08)70117-7 18424356 PMC11917918

[B23] Ingraham L. J. Chard F. Wood M. Mirsky A. F. (1988). An Hebrew language version of the Stroop test. Percept. Mot. Skills 67, 187–192. 10.2466/pms.1988.67.1.187 3211671

[B24] Ivnik R. J. Malec J. F. Smith G. E. Tangalos E. G. Petersen R. C. (1996). Neuropsychological tests' norms above age 55: COWAT, BNT, MAE token, WRAT-R reading, AMNART, STROOP, TMT, and JLO. Clin. Neuropsychol. 10, 262–278. 10.1080/13854049608406689

[B25] Jankovic J. McDermott M. Carter J. Gauthier S. Goetz C. Golbe L.. (1990). Parkinson Study Group. Variable expression of Parkinson's disease: a base-line analysis of DATATOP cohort. Neurology 40, 1529–1534. 2215943 10.1212/wnl.40.10.1529

[B26] Jensen A. R. Rohwer W. D. (1966). The Stroop Color-Word Test: a Review. Acta Psychol. 25, 36–93. 10.1016/0001-6918(66)90004-7 5328883

[B27] Kane M. J. Engle R. W. (2003). Working-memory capacity and the control of attention: the contributions of goal neglect, response competition, and task set to Stroop interference. J. Exp. Psychol. Gen. 132, 47–70. 10.1037/0096-3445.132.1.47 12656297

[B28] Kang C. Lee G. J. Yi D. McPherson S. Rogers S. Tingus K.. (2013). Normative data for healthy older adults and an abbreviated version of the Stroop test. Clin. Neuropsychol. 27, 276–289. 10.1080/13854046.2012.742930 23259830

[B29] Lansbergen M. M. Kenemans J. L. van Engeland H. (2007). Stroop interference and attention-deficit/hyperactivity disorder: a review and meta-analysis. Neuropsychology 21:251. 10.1037/0894-4105.21.2.251 17402825

[B30] Llinàs-Reglà J. Vilalta-Franch J. López-Pousa S. Calvó-Perxas L. Garre-Olmo J. (2013). Demographically adjusted norms for Catalan older adults on the Stroop Color and Word Test. Arch. Clin. Neuropsychol. 28, 282–296. 10.1093/arclin/act003 23380811

[B31] Lopez E. Salazar X. F. Villasenor T. Saucedo C. Pena R. (2013). Validez y datos normativos de las pruebas de nominación en personas con educación limitada, in Poster Presented at The Congress of the “Sociedad Lationoamericana de Neuropsicologıa” (Montreal, QC).

[B32] Lubrini G. Periañez J. A. Rios-Lago M. Viejo-Sobera R. Ayesa-Arriola R. Sanchez-Cubillo I.. (2014). Clinical Spanish norms of the Stroop test for traumatic brain injury and schizophrenia. Span. J. Psychol. 17:E96. 10.1017/sjp.2014.90 26055495

[B33] Lucas J. A. Ivnik R. J. Smith G. E. Ferman T. J. Willis F. B. Petersen R. C.. (2005). Mayo's older african americans normative studies: norms for boston naming test, controlled oral word association, category fluency, animal naming, token test, wrat-3 reading, trail making test, stroop test, and judgment of line orientation. Clin. Neuropsychol. 19, 243–269. 10.1080/13854040590945337 16019707

[B34] MacLeod C. M. Dunbar K. (1988). Training and Stroop-like interference: evidence for a continuum of automaticity. J. Exp. Psychol. Learn. Mem. Cogn. 14, 126–135. 10.1037/0278-7393.14.1.126 2963892

[B35] McDowd J. M. Oseas-Kreger D. M. Filion D. L. (1995). Inhibitory processes in cognition and aging, in Interference and Inhibition in Cognition, eds Dempster F. N. Brainerd C. J. (San Diego, CA: Academic Press), 363–400.

[B36] Mitrushina M. Boone K. B. Razani J. D'Elia L. F. (2005). Handbook of Normative Data for Neuropsychological Assessment. New York, NY: Oxford University Press.

[B37] Moering R. G. Schinka J. A. Mortimer J. A. Graves A. B. (2004). Normative data for elderly African Americans for the Stroop color and word test. Arch. Clin. Neuropsychol. 19, 61–71. 10.1093/arclin/19.1.61 14670380

[B38] Morrow S. A. (2013). Normative data for the stroop color word test for a north american population. Can. J. Neurol. Sci. 40, 842–847. 10.1017/S0317167100015997 24257227

[B39] Norman M. A. Moore D. J. Taylor M. Franklin D. Jr. Cysique L. Ake C.. (2011). Demographically corrected norms for African Americans and Caucasians on the hopkins verbal learning test–revised, brief visuospatial memory test–revised, stroop color and word test, and wisconsin card sorting test 64-card version. J. Clin. Exp. Neuropsychol. 33, 793–804. 10.1080/13803395.2011.559157 21547817 PMC3154384

[B40] Oliveira R. M. Mograbi D. C. Gabrig I. A. Charchat-Fichman H. (2016). Normative data and evidence of validity for the Rey Auditory Verbal Learning Test, Verbal Fluency Test, and Stroop Test with Brazilian children. Psychol. Neurosci. 9, 54–67. 10.1037/pne0000041

[B41] Peña-Casanova J. Qui-ones-Ubeda S. Gramunt-Fombuena N. Quintana M. Aguilar M. Molinuevo J. L.. (2009). Spanish multicenter normative studies (NEURONORMA Project): norms for the Stroop color-word interference test and the Tower of London-Drexel. Arch. Clin. Neuropsychol. 24, 413–429. 10.1093/arclin/acp043 19661108

[B42] Rivera D. Perrin P. B. Stevens L. F. Garza M. T. Weil C. Saracho C. P.. (2015). Stroop color-word interference test: normative data for the Latin American Spanish speaking adult population. Neurorehabilitation 37, 591–624. 10.3233/NRE-151281 26639926

[B43] Rognoni T. Casals-Coll M. Sánchez-Benavides G. Quintana M. Manero R. M. Calvo L.. (2013). Spanish normative studies in a young adult population (NEURONORMA young adults Project): norms for the Boston Naming Test and the Token Test. Neurología 28, 73–80. 10.1016/j.nrl.2012.02.009 22405213

[B44] Rosselli M. Ardila A. Santisi M. N. Arecco Mdel R. Salvatierra J. Conde A.. (2002). Stroop effect in Spanish–English bilinguals. J. Int. Neuropsychol. Soc. 8, 819–827. 10.1017/S1355617702860106 12240746

[B45] Seo E. H. Lee D. Y. Kim S. G. Kim K. W. Youn J. C. Jhoo J. H.. (2008). Normative study of the Stroop Color and Word Test in an educationally diverse elderly population. Int. J. Geriatr. Psychiatry 23, 1020–1027 10.1002/gps.2027 18425990

[B46] Shum D. H. K. McFarland K. A. Brain J. D. (1990). Construct validity of eight tests of attention: comparison of normal and closed head injured samples. Clin. Neuropsychol. 4, 151–162. 10.1080/13854049008401508

[B47] Stacy M. Jankovic J. (1992). Differential diagnosis of parkinson's disease and the parkinsonism plus syndrome. Neurol. Clin. 10, 341–359. 1584178

[B48] Steinberg B. A. Bieliauskas L. A. Smith G. E. Ivnik R. J. (2005). Mayo's older Americans normative studies: age-and IQ-adjusted norms for the trail-making test, the stroop test, and MAE controlled oral word association test. Clin. Neuropsychol. 19, 329–377. 10.1080/13854040590945210 16120535

[B49] Strauss E. Sherman E. M. Spreen O. (2006a). A Compendium of Neuropsychological Tests: Administration, Norms, and Commentary. Oxford: American Chemical Society.

[B50] Strauss E. Sherman E. M. S. Spreen O. (2006b). A Compendium of Neuropsychological Tests, 3rd Edn. New York, NY: Oxford University Press.

[B51] Strickland T. L. D'Elia L. F. James R. Stein R. (1997). Stroop color-word performance of African Americans. Clin. Neuropsychol. 11, 87–90. 10.1080/13854049708407034

[B52] Stroop J. R. (1935). Studies of interference in serial verbal reactions. J. Exp. Psychol. 18, 643–662. 10.1037/h0054651

[B53] Stuss D. T. Floden D. Alexander M. P. Levine B. Katz D. (2001). Stroop performance in focal lesion patients: dissociation of processes and frontal lobe lesion location. Neuropsychologia 39, 771–786. 10.1016/S0028-3932(01)00013-6 11369401

[B54] Swick D. Jovanovic J. (2002). Anterior cingulate cortex and the Stroop task: neuropsychological evidence for topographic specificity. Neuropsychologia 40, 1240–1253. 10.1016/S0028-3932(01)00226-3 11931927

[B55] Tremblay M. P. Potvin O. Belleville S. Bier N. Gagnon L. Blanchet S. (2016). The victoria stroop test: normative data in Quebec-French adults and elderly. Arch. Clin. Neuropsychol. 31, 926–933. 10.1093/arclin/acw029 PMC5859918 27246959

[B56] Trenerry M. R. Crosson B. DeBoe J. Leber W. R. (1989). Stroop Neuropsychological Screening Test. Odessa, FL: Psychological Assessment Resources.

[B57] Troyer A. K. Leach L. Strauss E. (2006). Aging and response inhibition: normative data for the Victoria Stroop Test. Aging Neuropsychol. Cogn. 13, 20–35. 10.1080/138255890968187 16766341

[B58] Valgimigli S. Padovani R. Budriesi C. Leone M. E. Lugli D. Nichelli P. (2010). The Stroop test: a normative Italian study on a paper version for clinical use. G. Ital. Psicol. 37, 945–956. 10.1421/33435

[B59] Van der Elst W. Van Boxtel M. P. Van Breukelen G. J. Jolles J. (2006). The Stroop Color-Word Test influence of age, sex, and education; and normative data for a large sample across the adult age range. Assessment 13, 62–79. 10.1177/1073191105283427 16443719

[B60] Vendrell P. Junqué C. Pujol J. Jurado M. A. Molet J. Grafman J. (1995). The role of prefrontal regions in the Stroop task. Neuropsychologia 33, 341–352. 10.1016/0028-3932(94)00116-7 7792000

[B61] Venneri A. Molinari M. A. Pentore R. Cotticelli B. Nichelli P. Caffarra P. (1992). Shortened Stroop color-word test: its application in normal aging and Alzheimer's disease. Neurobiol. Aging 13, S3–S4. 10.1016/0197-4580(92)90135-K

[B62] Waldrop-Valverde D. Ownby R. L. Jones D. L. Sharma S. Nehra R. Kumar A. M.. (2015). Neuropsychological test performance among healthy persons in northern India: development of normative data. J. Neurovirol. 21, 433–438. 10.1007/s13365-015-0332-4 25784168

[B63] Zajano M. J. Gorman A. (1986). Stroop interference as a function of percentage of congruent items. Percept. Mot. Skills 63, 1087–1096. 10.2466/pms.1986.63.3.1087

[B64] Zalonis I. Christidi F. Bonakis A. Kararizou E. Triantafyllou N. I. Paraskevas G.. (2009). The stroop effect in Greek healthy population: normative data for the Stroop Neuropsychological Screening Test. Arch. Clin. Neuropsychol. 24, 81–88. 10.1093/arclin/acp011 19395358

[B65] Zimmermann N. Cardoso C. D. O. Trentini C. M. Grassi-Oliveira R. Fonseca R. P. (2015). Brazilian preliminary norms and investigation of age and education effects on the Modified Wisconsin Card Sorting Test, Stroop Color and Word test and Digit Span test in adults. Dement. Neuropsychol. 9, 120–127. 10.1590/1980-57642015DN92000006 PMC5619350 29213953

